# DIFFERENCES IN SOCIAL CHARACTERISTICS AND HEALTH STATUS BY SEXUAL IDENTITY AMONG OLDER ADULTS: COMPARISON OF TWO COHORTS

**DOI:** 10.1093/geroni/igae098.0110

**Published:** 2024-12-31

**Authors:** James Iveniuk, Michelle Johns, Selena Zhong, Kristen Schilt

**Affiliations:** The National Opinion Research Center, Chicago, Illinois, United States; NORC at the University of Chicago, Chicago, Illinois, United States; NORC at the University of Chicago, Chicago, Illinois, United States; University of Chicago, Chicago, Illinois, United States

## Abstract

Sexual minority women experience poorer social, mental, and physical health than their heterosexual counterparts; yet limited work has been done to describe the scope of these health differences. Challenges to such analyses include a dearth of large-scale national surveys that collect sexual orientation data and small numbers of cases among those studies that do ask. To advance our understanding of the health and wellbeing of sexual minority women, we compare key indicators of health and wellbeing (e.g., diabetes, high blood pressure, asthma, depression, loneliness) by sexual identity in the National Social Life, Health, and Aging Project, a nationally representative study of older adults, and in SAMLAP, a nationally representative sample of older LGBT adults. We employ OLS and logistic regressions to assess how health outcomes differ between sexual minority women and heterosexual women in NSHAP as well as health differences among sexual minority women in SAMLAP. We controlled for age, marital status, race, education, and network size in our models. In NSHAP, we found that sexual minority women had higher depressive symptoms than heterosexual women but there were no other health differences between these two groups. In SAMLAP, bisexual women had higher odds of developing diabetes and more depressive symptoms compared to lesbian women. We will discuss our results within the context of the minority stress model which details the impact of social stigma on the health of sexual minority populations.

